# Testing support models for implementing an evidence-based digital intervention for alcohol use disorder: results of a pragmatic hybrid implementation-effectiveness trial

**DOI:** 10.21203/rs.3.rs-4004555/v1

**Published:** 2024-03-28

**Authors:** Andrew Quanbeck, Ming-Yuan Chih, Linda Park, Xiang Li, Qiang Xie, Alice Pulvermacher, Samantha Voelker, Rachel Lundwall, Katherine Eby, Bruce Barrett, Randy Brown

**Affiliations:** University of Wisconsin-Madison; University of Kentucky; University of Wisconsin-Madison; University of Wisconsin-Madison; University of Wisconsin-Madison; University of Wisconsin-Madison; University of Wisconsin-Madison; University of Wisconsin-Madison; UW Health; University of Wisconsin-Madison; University of Wisconsin Madison

## Abstract

This paper reports results of a hybrid effectiveness-implementation randomized trial that systematically varied levels of human oversight required to support implementation of a digital medicine intervention for persons with mild to moderate alcohol use disorder (AUD). Participants were randomly assigned to three groups representing possible digital health support models within a health system: self-monitored use (*n* = 185), peer-supported use (*n* = 186), or a clinically integrated model (*n* = 187). Across all three groups, percentage of risky drinking days dropped from 38.4% at baseline (95%CI [35.8%, 41%]) to 22.5% (19.5%, 25.5%) at 12 months. The clinically integrated group showed significant improvements in mental health quality of life compared to the self-monitoring group (p = 0.011). However, higher rates of attrition in the clinically integrated group warrants consideration in interpreting this result. Results suggest that making a self-guided digital intervention available to patients may be a viable option for health systems looking to promote alcohol risk reduction.

## INTRODUCTION

Alcohol use disorder (AUD) is a significant public health concern.^1^ AUD is a chronic, relapsing condition and is a leading cause of accidental death in the US.^2–10^ Excessive alcohol use was responsible for roughly 140,000 deaths per year according to data from the CDC, with an average of 3.6 million years of potential life lost annually.^11^ The rate of high-risk drinking has been increasing steadily in recent years, and the COVID-19 public health emergency increased those numbers substantially.^12^ In 2020, roughly 7% of US adults aged 18 and older had an AUD, but only 3% received treatment.^13^ Improving access to effective interventions is warranted for a disorder as pervasive as AUD. AUD can progress over the lifetime among at-risk individuals and can be conceptualized along a spectrum of risk, with appropriate interventions dependent on an individual’s risk of progressing to severe AUD.^14,15^

Accordingly, experts have suggested implementing harm-reduction approaches that have demonstrated improved outcomes for people with AUD.^15,16^ However, there is limited information on the effectiveness of interventions for the general population presenting with mild to moderate AUD. AUD prevention efforts could benefit from evidence-based treatment options that patients can access as part of primary healthcare.

Patient self-management is fundamental to addressing AUD because it allows individuals with risky use to initiate their own positive behavior change without specialty care.^17^ Smart technology and digital medicine apps have been increasingly used to help patients manage their chronic conditions,^18–21^ and digital medicine has demonstrated positive outcomes related to unhealthy alcohol use.^22–24^ With 85% of the US adult population owning a smartphone in 2021,^25^ digital medicine apps hold promise for the treatment and continuing care of patients with AUD because such systems can provide a widely accessible vehicle for both self-management and clinical monitoring.^26,27^ However, there has been little research on how best to implement digital medicine technologies for alcohol use (and other chronic diseases) to effectively reach populations that could benefit from them and to appropriately inform their ongoing health care. A key question relates to the role of human support (e.g., medical providers, health coaches, etc.) in enhancing the effectiveness of digital medicine apps.^28–30^

## METHODS

This study tested several pragmatic digital medicine support models that systematically varied the level and type of human support used to facilitate implementation of an evidence-based digital medicine intervention for AUD. The primary aim was to detect the effectiveness of each support model on self-reported risky drinking days (RDD: more than 4 standard drinks on any day for men under 65, more than 3 standard drinks for women and men over 65) and quality of life (QOL). We conducted a 12-month randomized controlled trial using a hybrid type 1 effectiveness-implementation study design to test the effectiveness of an intervention while exploring implementation aspects associated with varying digital medicine support models. Our rationale for this design is that a low-touch model is the least costly and easiest to implement. However, if active involvement of either peer support specialists or health coaches increases the intervention’s effectiveness, the additional cost may represent a worthwhile investment. Our null hypothesis was that adding human support in the form of peer mentoring and health coaching to self-guided use would have no additional effect on RDD and QOL (other secondary outcomes are reported separately).^31^ Our alternative hypotheses were that (1) adding group-oriented peer mentorship would increase effectiveness compared to the self-monitoring group and (2) adding 1:1 health coaching would increase effectiveness compared to the self-monitoring group.

### Theoretical and Empirical Foundations

This RCT used a digital medicine app called Tula (Sanskrit for “balance”). Tula was adapted from the Addiction-Comprehensive Health Enhancement Support System (A-CHESS)—one of the first digital medicine apps proven effective in an RCT of patients recovering from severe AUD after leaving a 90-day in-patient rehabilitation facility.^24^ These results were replicated when A-CHESS was shown to be effective in reducing heavy drinking in a 2022 study comparing telephone- and smartphone-based continuing care for AUD.^32^ A-CHESS has also been tested across a range of other populations.^33–37^

The theoretical framework supporting the A-CHESS app is self-determination theory, which asserts that meeting three fundamental psychological needs (competency, relatedness, and autonomous motivation) produces intrinsic motivation for behavior change.^38^

### Eligibility Criteria

Eligibility criteria are listed in Table 1. To maintain anonymity, all participants were identified by usernames they created while setting up their accounts.

### Setting

Study participants were recruited within the geographic catchment area of an integrated health system serving more than 700,000 patients each year in Wisconsin and the Upper Midwest. Individuals interested in the study used a QR code or a link from the study website to complete a secure, web-based screening survey. IP addresses and location verification were used to confirm that research participants lived within the health system’s catchment area.

### Intervention and Support Models

Tula is designed to address a broad spectrum of issues related to alcohol misuse likely to be found in the general population. Unlike A-CHESS, which focuses on abstinence as part of recovery from severe AUD, Tula focuses on reducing alcohol consumption for individuals with mild to moderate AUD. Tula is built upon the Whole Health Model, which focuses on “what matters to me” as a whole person (and not just the disease- “what’s the matter with me?”).^31,35^ Tula places alcohol use within the context of integrative medicine, an adaptation made based on input from partnering healthcare stakeholders.^31^ Tools and services included in Tula are listed in Table 2.

### Digital Medicine Support Models: Study Arms

**Figure 2:**
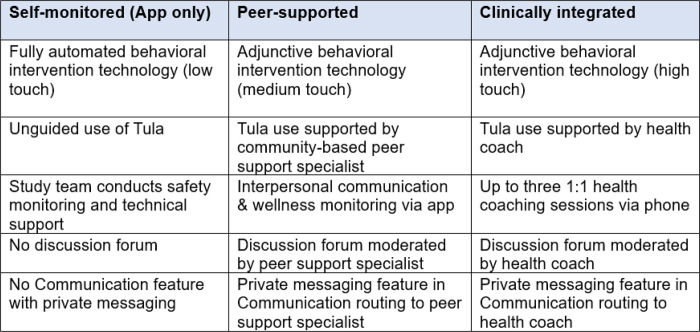
Study design. Adapted from: Hermes, E. D., Lyon, A. R., Schueller, S. M., & Glass, J. E. (2019). Measuring the implementation of behavioral intervention technologies: recharacterization of established outcomes. *Journal of medical Internet research, 21*(1), e11752.

The study was designed to examine the clinical integration of digital medicine, as described by Hermes et al.,^39^ where we compared a fully automated support model with two human-guided support models, differentiated by graduated levels of external support. While all participants had access to the basic content in Tula, the three study arms varied by the level of human touch (self-monitored “low touch,” peer-supported “medium touch,” and clinically integrated-“high touch”). See Fig. 2.

#### Self-monitored (SM) Group (low touch).

Participants in this group used the app on their own, like most commercially available health apps. They had no access to a discussion forum or private messaging nor any connection to an external support system (i.e., peer support specialist or health coach). Participants could communicate with the study team via email, phone, or a messaging functioning built into Tula. However, communication was limited to questions about the study and receiving tech support.

#### Peer-supported (PS) Group (medium touch).

Participants in this group had access to social support from others in the same study arm via Tula’s discussion forum and to certified peer specialists who worked for a community-based non-profit organization that has historically partnered with the health system to provide peer-mentoring services for patients with substance use disorders. Peer specialists are certified recovery coaches who have lived experience with substance use and/or mental health issues. They were not assigned to individual participants but were available to any participant who reached out via Tula’s private messaging feature. They also facilitated and moderated discussion forums and encouraged participants to utilize Tula’s resources.

#### Clinically Integrated (CI) Group (high touch).

Participants in this group worked one-on-one with health coaches employed within the healthcare system and had access to a discussion forum with other participants in their study arm. Health coaches are trained and certified in behavior change theories, motivational strategies, and health education. They worked with participants 1:1 to set goals for a healthier lifestyle through reduced alcohol consumption. Health coaches engaged with participants via Tula’s private messaging feature. Participants were offered three 1:1 health coaching sessions (via phone).

### Participant Recruitment, Retention and Timeline

We used a three-pronged recruitment strategy encompassing clinical settings, community-based organizations, and public media. We enlisted clinical study champions (primary care providers, behavioral health specialists, etc.) to provide information to potentially eligible patients. We also engaged local leaders from underrepresented communities to promote the study in ways that invite inclusion of diverse perspectives. Lastly, we used targeted digital, television, and print media to promote the study broadly.

All participants who completed the eligibility screening survey received a $10 digital gift code. Study incentives were built into the first 12 weekly check-in surveys (as part of the self-monitoring feature of Tula) and four quarterly follow-up surveys at months 3, 6, 9, and 12. The incentives were distributed monthly in the form of digital gift codes sent via the message feature in Tula. By the end of the 12-month study period, participants could earn up to US $250 in digital gift codes for data submission.

Eligible participants were given a 72-hour window to download the app, complete a baseline survey and schedule a phone call with research staff for enrollment. Recruitment lasted from March 2020 – September 2023 with the last participant completing their 12-month intervention in September 2024.

### Patient Randomization

A sequence of randomized assignments for each group was generated by the project statistician using the block randomization procedure in the Power Analysis and Statistical Software package.^40^ Randomization was stratified on participant-reported biological sex (male/female) and alcohol use severity (mild/moderate) based on DSM-5 score (mild = 2–3 and moderate = 4–6),^41^ calculated from participants’ responses to the screening survey. The randomization list was masked and placed in a protected spreadsheet. The randomization process involved a phone call between a member of the study team and the participant. When the study arm assignment was unmasked, a study ID was entered into the next available placement in the randomization sequence. While still on the call, the study team member configured the participant’s permissions on the app based on their group assignment and reviewed a second informed consent specific to that study arm. Once randomized, participants engaged in a 3-month active intervention period with a 9-month follow-up period.

### Blinding

Due to pragmatic and ethical considerations, the randomization assignment was not blind to either implementers or participants.

### Outcomes

#### Demographics and Severity.

Participants reported biological sex and their AUD severity (mild or moderate; those with severe AUD were excluded) in the screening survey, which were used as strata for randomization. Participants reported additional demographics, including age, race/ethnicity, education, and income in the baseline survey.

#### Primary Outcomes.

The primary outcomes were RDD and QOL. RDD were measured using the 7-day timeline follow-back survey.^42,43^ Participants were asked how many days they had more than 4 standard drinks (for men under 65) or more than 3 standard drinks (for women and for men over 65 years) on any single day in the last 7 days. The definition of a standard drink provided in the survey is “One drink = one shot of hard liquor (1.5 oz.), a 12-ounce can or bottle of beer, or a 5-ounce glass of wine.” To allow for comparison with other studies using varying recall periods, we converted the number of RDD to the percentage of RDD (PRDD) by dividing the number of RDD by 7 (days).

Patient-reported QOL was measured using the four global physical health and four global mental health items in the Patient-Reported Outcome Measurement Information System (PROMIS) global health short form (SF10 ver.1.2).^44^ The raw scores for physical and mental health were converted to T-scores using the PROMIS scoring manual (p.16).^44^ T-scores are standardized scores that can be compared to the US general population that has the mean QOL score of 50 with a standard deviation of 10.

Participants received automated reminders to complete surveys in the app. Survey data were stored on a secure server at the University of Wisconsin.

### Sample Size

The study was designed to detect differences in two primary outcomes (RDD and the QOL) among three study arms across a 12-month period. Sufficient power (1-β = .80, multiple comparison adjusted α = .00833, 2-tailed) to detect a conservative effect size of Cohen’s d = 0.25 with four repeated measurements and a first-order autoregressive covariance structure (correlation ρ = 0.2) required approximately 182 participants per study arm (546 total), assuming 28% attrition.^32^ An effect size of 0.25 equated to a difference of approximately 0.24 RDD per week, 2.18 points for QOL-Physical Health, and 2.03 points for QOL-Mental Health.^44^

### Statistical Methods

#### Descriptive Analysis.

We conducted descriptive analysis for demographic, utilization, and outcome variables across all three study arms. Group comparisons were conducted using chi-squared tests for categorical variables and t-tests for continuous variables. All statistical tests were two-tailed with α = 0.05 and conducted using SPSS 29.

#### Primary Analysis.

We constructed a longitudinal model of the outcome measures at months 3, 6, 9, and 12 after randomization using the General Linear Mixed Model (GLMM) with a random intercept and an auto-regression covariance structure for repeated measures. The stratification variables and the baseline values of the outcomes were included as covariates, with a separate model for each primary outcome. The primary analysis is the group fixed effects and pairwise comparisons between study arms. Other significant fixed effects and pair-wise comparisons were reported. The Sidak method was used to adjust p-values in pairwise comparisons.^45^ Because the value of PRDD has a positive skewness, we used the square root transformed PRDD, consistent with prior analyses of A-CHESS.^32^ Participants who could not be reached for follow-up surveys were analyzed following the intention to treat principle. Missing outcomes data were considered missing at random. Other secondary analyses (mediation, economic, and qualitative)^31^ will be reported separately.

#### Ancillary Analyses.

Sensitivity analyses are included to examine model assumptions regarding equivalence of groups at baseline and to account for differential attrition (see **Technical Appendix**).

### Important Changes after Trial Commencement

Originally, in-person health coaching was available but due to COVID-19, coaching was only offered via telephone. Coaches had access to participant phone numbers but only knew participants by their usernames.

### Ethics

This protocol was approved by the University of Wisconsin Health Sciences Minimal Risk Institutional Review Board and prospectively registered at clinicaltrials.gov (NCT04011644).

## RESULTS

### Study Flow and Survey Completion

**Figure 3:**
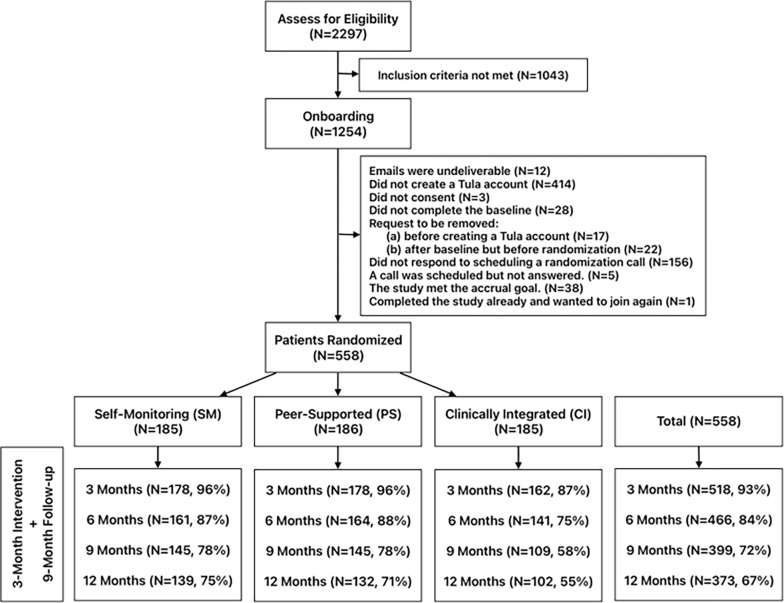
Study Flow Diagram. Participants who contacted the research team to drop out of the study: SM=0, PS=1, CI=9.

Among 2,297 individuals who completed the eligibility screening, 1,254 (55%) met the screening criteria and were invited to join the onboarding process; 558 (44%) completed the onboarding process and were randomized (Fig. 3). Two main reasons for those excluded during onboarding (n = 696) were not creating a Tula account (n = 414 or 59%) and not scheduling a randomization call (n = 156 or 22%). The completion rate of the follow-up surveys was overall 78% (1,756/2,232), ranging from 93% at the end of month 3 to 67% at month 12. The mean number of surveys completed per person across groups was 3.14 out of 4 follow-up surveys, with SD of 1.31. The CI group participants (M = 2.75, SE = 0.11) completed fewer follow-up surveys than the SM or PS group participants (Wald Chi-square (2) = 14.179, p < 0.001).

### Characteristics of the Study Population

Table 3 shows baseline characteristics of the study population. The majority (91%) of the study population was white, slightly higher than the proportion of white patients in the overall patient population in the health system (82%). The proportion of black participants enrolled in the study was roughly the same as their representation in the health system population (4.8%) and the Madison, WI metropolitan area. Females were overrepresented, comprising almost 2/3 of the study population. The population was highly educated, reflecting that Madison, WI, is the 5th most educated city in the US. Except for education level, all other demographics and outcomes at baseline were similar between groups. The CI group had a slightly higher percentage with a high school degree/GED as their highest education level but a slightly lower percentage of those who completed a vocational or associate degree. The PS group had a slightly lower percentage with masters’ degrees.

### Intervention Utilization

**Figure 4:**
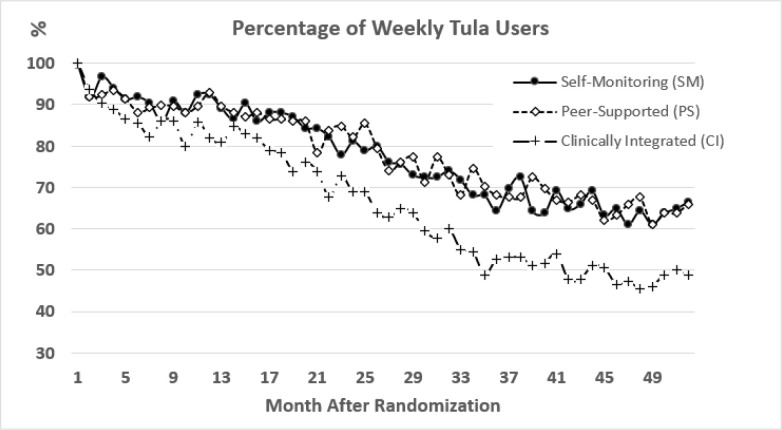
Percentage of weekly Tula users over time

Figure 4 depicts the percentage of participants who logged into Tula at least once during each study week. Use declined over time, most notably among the CI participants. Participants clicked an average of 806 links/pages in the Tula app during the 12-month study period with a wide range from 6 to 4,844 pages. An average of 11.2 out of 12 weekly check-in surveys were submitted during the 3-month intervention period (with incentives), and 28.3 out of 40 weekly surveys were submitted during the rest of the 9-month follow-up period (without incentives). The PS and CI group participants could read and post messages on Tula discussion forums. Most PS and CI participants (96%, 178/186 and 97%, 182/187, respectively) read Tula discussion forum messages while 96 (52%) PS and 52 (28%) CI participants posted one or more messages. Health coaching visits were utilized by 125 (67%) CI participants with a mean number of 1.7 visits and a SD of 1.3.

### Descriptive Analysis- Primary Outcomes

**Figure 5:**
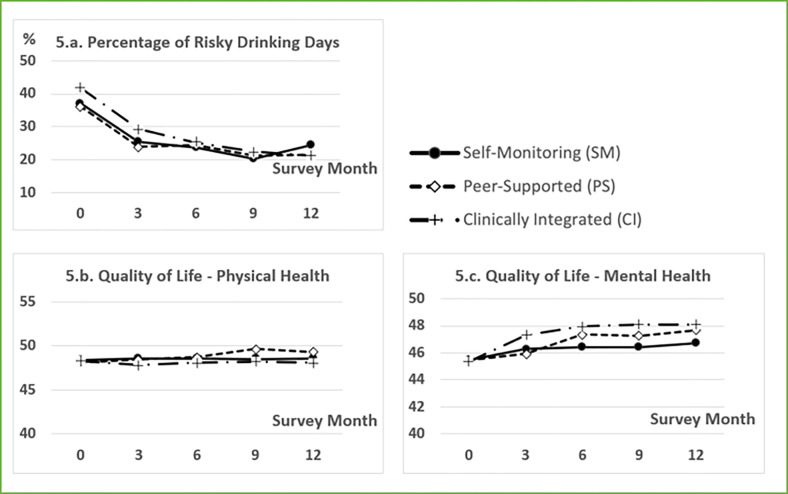
Outcomes over time. Data points in these three charts are group means. The standard deviations are listed in Table 4.

Mean values of primary outcomes from baseline to month 12 for all three groups are listed in Table 4 and visualized in Fig. 5. Absolute and relative risk reductions between outcomes at baseline to 12 months are listed in Table 4. An overall decline in PRDD over time—from an average 38.4 PRDD (95%CI [35.8, 41]) at baseline to 22.5 PRDD (19.5, 25.5) at month 12, about a reduction of 15.9 PRDD or a 41% reduction in PRDD—was observed across all the study groups. However, rates of PRDD in the SM group bounced back slightly at month 12, while the PS group remained the same, and the CI group continued to decrease. The physical health scores showed little change except for a slight improvement for the PS group from month 6 (48.7) to month 9 (49.6). In the mental health domain, all three groups showed slight improvements in mental health with more noticeable improvement for the CI (5.9%) and PS groups (4.8%) from baseline to month 12 compared to the SM group (2.6%).

### Primary Statistical Analysis

#### Percentage Risky Drinking Days (PRDD).

The results of the **GLMM** analysis are listed in Table 5. There was no group effect on PRDD (F(2,1729) = 0.375, p = 0.688). However, a significant overall time effect (F(2,1729) = 6.142, p < 0.001) and a trending group by time effect (F(6, 1729) = 1.936, p = 0.072) were found. The overall PRDD declined significantly from month 3 to months 9 and 12 (p < 0.001 and p = 0.005, respectively). The pairwise comparisons for the group by time effect showed that PRDD had a significant reduction from month 3 to months 9 and 12 among the CI group participants (F(3,1729) = 6.773, p < 0.001) but not in the other two groups (SM: F(3,1729) = 2.124, p = 0.095; PS: F(3,1729) = 0.474, P = 0.7).

#### Quality of Life-Physical Health (QOL-PH).

No group effect (F(2,1728) = 1.345, p = 0.261) was found in the mixed model analysis for QOL-PH. The overall time effect was significant (F(3, 1728) = 2.856, p = 0.036) but the group by time effect was not (F(6,1728) = 0.695, p = 0.654). The pairwise comparisons between the time points and the group by time interactions were not statistically significant.

#### Quality of Life-Mental Health (QoL-MH).

An overall group effect (F(2,1728) = 4.277, p = 0.014) and time effect (F(3,1728) = 4.597, p = 0.003) were found for QoL-MH. The group by time effect was not significant (F(6,1728) = 0.984, p = 0.434). The pair-wise contrasts showed that the CI participants overall reported a better level of mental health (p = 0.011) than the SM group. The significant group difference was also found at 9 months (F(2,1727) = 3.989,p = 0.019) with a trending significance at 3 months (F(2,1728) = 2.862, p = 0.057) and 12 months (F(2,1728) = 2.644, p = 0.071). The overall QoL-MH across the three groups was found to improve significantly from 3 to 6 months and 3 to 12 months (p = 0.006 and 0.023, respectively). The PS participants reported significant improvement in QOL-MH (F(3,1728) = 4.681, p = 0.003) specifically from 3 months to 6 and 12 months (p = 0.01 and 0.015, respectively).

## DISCUSSION

### Summary of Results

This hybrid implementation-effectiveness trial systematically varied the degree of human touch offered to support the implementation of an evidence-based digital health app for alcohol use reduction in participants with mild to moderate AUD. All three randomized groups had reductions in alcohol use—from 38.4% (95%CI [35.8%, 41%]) at baseline to 22.5% (19.5%, 25.5%) at 12 months, representing a statistically significant time effect. The CI group showed a significant reduction in alcohol use from 3 to 9 and 12 months (p < 0.001 and p = 0.005 respectively) while the other two groups did not. There were no statistically significant differences in PRDD reduction between the SM, PS, and CI groups. The clinical and societal significance of a 16 percentage point reduction (41% relative reduction) in PRDD is difficult to gauge directly. For context, the CDC estimates that excessive alcohol use is responsible for more than 140,000 annual deaths and an economic cost of $249 billion.^11^ AUD is a chronic, relapsing and remitting condition that can worsen over the lifespan, and arresting problematic use across a broad base of the population before alcohol-related complications manifest could potentially have significant population health benefits.

Concerning the primary outcome of QOL, the null hypothesis was not rejected, with the SM group showing similar effectiveness as the PS and CI groups. The CI group showed significant improvements in mental health QOL scores compared to the SM group (p = 0.011). However, higher rates of attrition in the CI group warrants caution in interpreting the results (see **Technical Appendix).** It appears that younger persons dropped out at a higher rate than older participants, and a pattern mixture model conducted via sensitivity analysis (as opposed to the base case assumption of data missing at random) negated the positive effect on mental health quality of life in the CI group.

### Comparison with Prior Work

There has been little published research on digital medicine interventions targeted for this population. Crane et al. (2018) examined the Drink Less app in the U.K., which enrolled 672 participants.^46^ In that study, only 179 (27%) participants completed the primary outcome measure, and no main effects on alcohol use were found. The literature suggests that systematic tracking of alcohol use and mindful awareness of drinking patterns can serve as effective interventions *per se*,^47,48^ and this type of functionality was supported by all three groups, including the self-monitoring group.

With 558 participants, this study is the largest controlled trial featuring a version of A-CHESS. It is essential to highlight differences in the current study population compared to prior studies of A-CHESS.^24,32^ In contrast to prior studies that enrolled participants in recovery for severe AUD, our study participants had mild to moderate AUD. Connection with peers has been integral to prior versions of A-CHESS, and this dynamic was modeled in the PS group via peer mentorship and the discussion forum. However, peer mentoring had minimal additional effect compared to self-monitored use in this study, perhaps because isolation is not as prevalent as it often becomes for persons with more severe AUD.

### Strengths and Limitations

With 558 randomized participants and an overall response rate of 78%, this study was among the most expansive and rigorously conducted digital health trials for alcohol use reduction. Follow-up rates were high compared to norms in digital health research, where a 2022 systematic review found median completion rates of only 48% across 37 studies (a 78% response rate would correspond to the first quartile).^49^ Follow up rates did vary by group, however. The research team took note of higher dropout rates in the CI group early in the trial and made repeated efforts to contact participants to increase follow-up, with limited success. The study team is conducting qualitative interviews with participants to further examine their experience with health coaching. Early participant reports signal that 1:1 health coaching over the phone was experienced differently than using an app independently, and differential drop-out rates between those randomized to health coaching vs. the other 2 groups suggest that 1:1 health coaching may not be acceptable to some individuals with mild to moderate AUD.

This study focused on implementation of digital health in a primary healthcare system and was intentionally pragmatic vs. explanatory, as outlined by PRECIS-2 criteria.^50^ Pragmatic study design decisions were made in partnership with health system leadership, including self-report of alcohol use without biomarker verification, unblinded assignment for implementers and participants, and implementation by health care and community-based practitioners vs. research staff. Importantly, as a type 1 hybrid effectiveness-implementation trial, the study did not include a pure control group. Doing so would require a “sham device,” which was deemed impractical given the aims of the study: no plausible placebo digital medicine support model for AUD could be envisioned that would be acceptable to the health system. Improvements in PRDD were about the same in all 3 groups, so there is no direct evidence that Tula was the cause of the improvements. Participation in research can have effects, regardless of the specific interventions tested. Other factors may explain alcohol use reductions observed (e.g., temporal trends, Hawthorne effect). At least two counter-arguments are notable, however: (1) The study took place during the COVID pandemic, where drinking rates significantly increased nationwide and in the state of Wisconsin in particular^51^ and (2) while alcohol use and QOL were assessed using the same survey at the same time points, no corresponding improvement in QOL was observed to mirror reductions in RDD.

Prior research suggests that people who use complementary and alternative medicine interventions tend to be female, of middle age, and have more education.^52^ Our sample reflects this. Our use of convenience sampling may limit generalizability to other populations and highlights the need to actively engage communities to keep health equity concerns at the forefront of digital health implementation research.^53^

### Implications

The results suggest that a population with mild to moderate AUD may reduce alcohol use through self-directed use of a digital health app. Paying mindful attention to alcohol use may be the first step in contemplating and addressing problematic habits and patterns of use that may lead to future problems. While adding peer support or 1:1 health coaching did not clearly yield more effectiveness in alcohol use reduction, incorporating patient preferences for using digital health apps may be worth considering when choosing digital medicine support models, particularly in light of the mental health improvements observed in the CI group. Additionally, the more intensive nature of the CI intervention may have represented treatment over-matching for persons with mild/moderate AUD. Choice in selecting an intervention has been shown to improve outcomes in other areas of research, such as heart disease.^54^ Thus, one possible implication for health systems would be to consider offering a self-guided version of the app with an option for supplemental health coaching for individuals who desire a personal connection or additional support.

## CONCLUSION

The trial results suggest that self-guided use may be as effective as more intensive digital health support models guided by peer mentors and health coaches in a population with mild to moderate AUD. A self-guided support model is certainly less expensive and simpler for health systems to adopt than human-guided support models, and the differential attrition in the CI group suggests that persons with mild to moderate alcohol use disorders may value autonomy and anonymity over personalized and more intensive health coaching in addressing mild to moderate AUD. In conclusion, making an evidence-based digital health intervention focused on wellness, mindful awareness, and prevention towards AUD progression may be a viable option for health systems looking to promote evidence-based interventions for AUD.

## Figures and Tables

**Figure 1:**
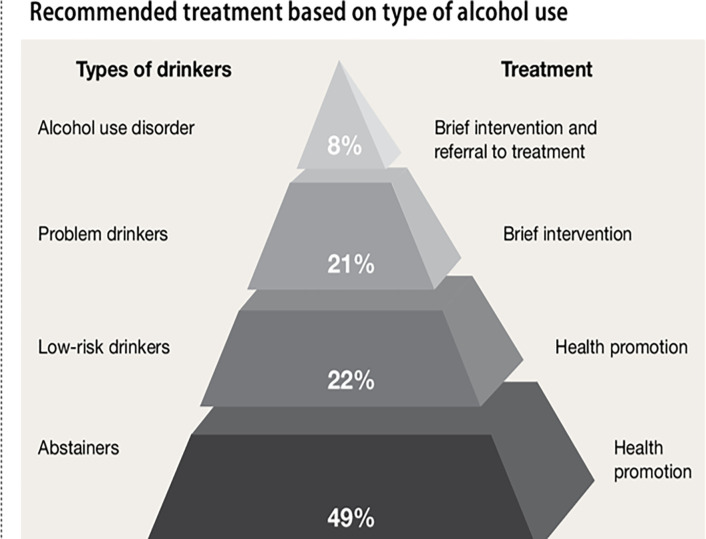
Spectrum of alcohol use. Adapted from: Goulding, E., & Fleming, M. (2011). Strategies to reduce alcohol use in problem drinkers. *Current Psychiatry, 10*(11), 30.

**Table 1:** Patient inclusion and exclusion criteria

Inclusion criteria	Exclusion criteria
21 years old or older	Reports symptoms consistent with severe AUD during screen (6 or more of 11 DSM-5 criteria)**
Wants to reduce drinking	Active psychotic disorder diagnosis
Owns a smartphone and willing to download and use the Tula app	Acute medical problem requiring immediate hospitalization
Lives within the healthcare system service area	Terminal illness

Meets at least one of the following criteria:

• AUDIT^55^ screening score 8+, OR

• Responds “yes” to at least 2 questions on the AUD DSM-5+,^41^ OR

• Reports moderate- to high-risk drinking patterns*

∘ more than 3 drinks on any single day and more than 7 drinks per week (women)

∘ more than 4 drinks on any single day and more than 14 drinks per week (men)

**Table 2:** Tula content and tools

Feature	Description
Thought of the Day	Daily inspirational quotes intended to motivate and engage participants.
Whole Health	Information and tools to improve one’s whole health: What is Whole Health; Circle of Health; Self-Care; Mindful Awareness; Whole Health Resources; Personal Health Inventory
Motivation	Users can record in words and photos their reasons for wanting to work on their drinking and wellness
Tracker *	The Tracker allows users to set and review goals, track their progress, and record their health and wellness patterns related to their quality of life (e.g., mood, sleep, social support).
Communication**	Users can send and receive private messages with other Tula members and can access in-app discussion forums with other members of their group.
Information	A content and resource library organized around the Circle of Health’s eight domains of self-care: Working Your Body, Sleep & Recharge, Food & Drink, Personal Development, Relationships, Mood & Mindset, Surroundings, and the Power of the Mind.
Relaxation	Relaxation techniques, audio recordings for guided meditation, and binaural beats.
Strategies	Tips for reducing drinking, cognitive behavioral therapy, and goal setting.
Gratitude	A daily prompt to reflect on gratitude.

* Tracker feature accessed by all participants but is also monitored by the health coaches in the clinically integrated group as part of goal setting.

** Communication features are limited in the self-monitored group.

**Table 3:** Baseline characteristics of the study population

Characteristic	Self-monitored (n = 185)	Peer-supported (n = 186)	Clinically integrated (n = 187)	Total (n = 558)
**Age** (M, SD)	(42.8, 12.9)	(42.8, 12.9)	(42.6, 12.9)	(42.8, 12.9)
**Female** (N, %)	(121, 65.4%)	(122, 65.6%)	(123, 65.8%)	(366, 65.6%)
**Race** (N, %)*				
White	(170, 91.9%)	(169, 90.9%)	(171, 91.4%)	(510, 91.4%)
Black	(7, 3.8%)	(10, 5.4%)	(10, 5.3%)	(27, 4.8%)
AI/NA	(2, 1.1%)	(2, 1.1 %)	(1, 0.5%)	(5, 0.9%)
Asian	(5, 2.7%)	(3, 1.6%)	(4, 2.1 %)	(12, 2.2%)
NH/PI	(0, 0%)	(1, 0.5%)	(1, 0.5%)	(2, 0.4%)
Other	(1, 0.5%)	(4, 2.2%)	(1, 0.5%)	(6, 1.1%)
**Hispanic or Latino** (N, %)	(4, 2.2%)	(6, 3.2%)	(4, 2.1 %)	(14, 2.5%)
**Education** (N, %)				
<High School	(0, 0%)	(2, 1.1 %)	(1, 0,5%)	(3, 0.5%)
HS or GED	(16, 8.6%)	(19, 10.2%)	(32, 17.1%)	(67, 12%)
Vocation or Associate	(35, 18.9%)	(34, 18.3%)	(16, 8.6%)	(85, 15.2%)
Bachelors	(76, 41.1 %)	(83, 44.6%)	(76, 40.6%)	(235, 42.1%)
Masters	(44, 23.8%)	(29, 15.6%)	(48, 25.7%)	(121, 21.7%)
Doctorate	(14, 7.6%)	(19, 10.2%)	(14, 7.5%)	(47, 8.4%)
**Marital status** (N, %)				
Married	(104, 56.2%)	(95, 51.1%)	(102, 54.5%)	(301, 53.9%)
Widowed	(3, 1.6%)	(3, 1.6%)	(4, 2.1 %)	(10, 1.8%)
Divorced	(16, 8.6%)	(22, 11.8%)	(17, 9.1%)	(55, 9.9%)
Separated	(2, 1.1%)	(3, 1.6%)	(1, 0.5%)	(6, 1.1%)
Never Married	(35, 18.9%)	(42, 22.6%)	(32, 17.1%)	(109, 19.5%)
Living with a partner	(24, 13%)	(19, 10.2%)	(30, 16%)	(73, 13.1%)
Refused	(0, 0%)	(0, 0%)	(1, 0.5%)	(1, 0.2%)
Don’t know	(1, 0.5%)	(2, 1.1 %)	(0, 0%)	(3, 0.5%)
**Severity-mild** (N, %)	(118, 63.8%)	(120, 64.5%)	(118, 63.1%)	(356, 63.8%)

**Notes:** Abbreviations: AI/NA: American Indian/Native American, NH/PI: Native Hawaiian/Pacific Islander, HS: High School, GED: General Educational Development, M: Mean, SD: Standard Deviation, N: Number, %: Percentage, QOL: Quality of Life.

* The numbers in race added within groups were over 100% because some people reported multiple races.

**Table 4:** Outcomes of the study population

Characteristic	Self-monitored		Peer-supported		Clinically integrated		Total	
	n	(M, SD)	n	(M, SD)	n	(M, SD)	n	(M, SD)
**Percentage of risky drinking days, PRDD**								
Baseline	185	(37.1, 30.6)	186	(36.2, 31.2)	186	(41.9, 31.4)	557	(38.4, 31.1)
3 Months	178	(25.5, 28.2)	178	(23.8, 30.1)	162	(29.4, 28.2)	518	(26.1, 28.9)
6 Months	161	(23.6, 27.3)	162	(24.3, 30.1)	139	(25.3, 31.2)	462	(24.4, 29.4)
9 Months	145	(20.3, 24.6)	145	(21.2, 27.7)	109	(22.4, 27.4)	399	(21.2, 26.5)
12 Months	139	(24.4, 28.2)	130	(21.4, 28.9)	100	(21.3, 30.4)	369	(22.5, 29)
ARR or RRR*	−12.7 or −34.2%		−14.8 or −40.9%		−20.6 or −49.2%		−15.9 or −41.4%	
**Quality of Life - Physical Health, QOL-PH** *								
Baseline	185	(48.4, 6.3)	186	(48.1, 6.7)	186	(48.3, 6.5)	557	(48.3, 6.5)
3 Months	178	(48.6, 6.6)	177	(48.4, 6.6)	162	(47.8, 6.7)	517	(48.3, 6.6)
6 Months	161	(48.6, 6.4)	162	(48.7, 7.2)	139	(48.1, 7.2)	462	(48.5, 6.9)
9 Months	145	(48.5, 6.3)	145	(49.6, 6.1)	109	(48.1, 7.4)	399	(48.8, 6.6)
12 Months	139	(48.6, 6.8)	130	(49.3, 6.4)	100	(48.1, 7.5)	369	(48.6, 6.9)
ARR or RRR*	−0.2 or 0.4%		1.2 or 2.5%		−0.2 or −0.4%		0.3 or 0.6%	
**Quality of Life - Mental Health, QOL-MH** *								
Baseline	185	(45.5, 6.9)	186	(45,5, 7.5)	186	(45.4, 6.9)	557	(45.4, 7.1)
3 Months	178	(46.3, 7)	177	(45,9, 7.3)	162	(47.3, 7.1)	517	(46.5, 7.1)
6 Months	161	(46.4, 7.1)	162	(47.4, 7.5)	139	(48, 7.5)	462	(47.2, 7.4)
9 Months	145	(46.4, 7.2)	145	(47.3, 7.2)	109	(48.1, 7.2)	399	(47.2, 7.2)
12 Months	139	(46.7, 8.3)	130	(47.7, 8.3)	100	(48.1, 7)	369	(47.4, 8)
ARR or RRR**	1.2, 2.6%		2.2, 4.8%		2.7, 5.9%		1.8, 4%	

Notes: Abbreviations: M: Mean, SD: Standard Deviation, n: Sample size, ARR: Absolute Risk Reduction, RRR: Relative Risk Reduction.

* QOL measures are T-scores.

** ARR and RRR were calculated between values in baseline and 12 months.

**Table 5:** Treatment and Time Contrast on Primary Outcomes

Outcome	Contrast*	Estimate (95%CI)	SE	df	P
PRDD**	PS-SM	−0.205 (−0.784, 0.374)	0.242	1729	0.783
	CI-SM	−0.145 (−0.75, 0.46)	0.253	1729	0.918
	CI-PS	0.059 (−0.546, 0.665)	0.253	1729	0.994
	12M-3M	−0.484 (−0.867, −0.101)	0.145	1729	0.005
	9M-3M	−0.557 (−0.928 −0.185)	0.141	1729	< 0.001
	CI: 12M-3M	−1.149 (−1.868, −0.43)	0.273	1729	< 0.001
	CI: 9M-3M	−0.884 (−1.578, −0.189)	0.264	1729	0.005
QoL-PH	PS-SM	0.339 (−0.592, 1.269)	0.389	1728	0.767
	CI-SM	−0.326 (−1.297, 0.644)	0.406	1728	0.806
	CI-PS	−0.665 (−1.636, 0.306)	0.406	1728	0.275
QoL-MH	PS-SM	0.519 (−0.701, 1.739)	0.510	1728	0.670
	CI-SM	1.540 (0.268, 2.811)	0.532	1728	0.011
	CI-PS	1.020 (−0.252, 2.293)	0.532	1728	0.157
	12M-3M	0.894 (0.078, 1.710)	0.310	1728	0.023
	6M-3M	0.814 (0.165, 1.463)	0.246	1728	0.006
	6M: CI-SM	1.587 (0.002, 3.172)	0.663	1728	0.050
	9M: CI-SM	1.972 (0.277, 3.667)	0.709	1728	0.016
	PS: 12M-3M	1.569 (0.203, 2.934)	0.518	1728	0.015
	PS: 6M-3M	1.309 (0.214, 2.405)	0.416	1728	0.010

**Notes:** Abbreviations: M: Month, PRDD: Percentages of risky drinking days, QoL-PH: Quality of life-Physical health, QoL-MH: Quality of life-Mental health, SM: Self-monitoring, PS: Peer-supported, CI: Clinically integrated.

* Treatment contrasts controlled for alcohol drinking severity at screening, biological sex, and outcomes at baseline. Contrasts for the main group comparisons were included. For the time and the group by time effects, only the significant level of pairwise comparisons lower than 0.1 were listed.

** PRDD is a square-root transformed variable.

## Data Availability

The SPSS dataset, containing de-identified data, and the SPSS syntax file, used to generate the results in this paper, are both available upon request sent to Dr. Andrew Quanbeck.
